# Determinants of contraceptive use among postpartum women in a county hospital in rural KENYA

**DOI:** 10.1186/s12889-017-4510-6

**Published:** 2017-06-29

**Authors:** Rose Jalang’o, Faith Thuita, Sammy O. Barasa, Peter Njoroge

**Affiliations:** 1 Division of Family Health, National Vaccines and Immunization Program, Ministry of Health , P. O. Box 43319–00100, Nairobi, Kenya; 2 Department of Nursing, Kenya Medical Training College, Chuka Campus, P. O. Box 641-6400, Chuka, Kenya; 3 Public Health Nutrition, School of Public Health, University of Nairobi, P. O. Box 19676 – 00200, Nairobi, Kenya; 4 Maternal and Child Health, School of Public Health, University of Nairobi, P.O. Box 19676 – 00200, Nairobi, Kenya

**Keywords:** Postpartum women, Contraceptive use, Determinants, Family planning, Kenya

## Abstract

**Background:**

There is a high unmet need for limiting and spacing child births during the postpartum period. Given the consequences of closely spaced births, and the benefits of longer pregnancy intervals, targeted activities are needed to reach this population of postpartum women. Our objective was to establish the determinants of contraceptive uptake among postpartum women in a county referral hospital in rural Kenya.

**Methods:**

Sample was taken based on a mixed method approach that included both quantitative and qualitative methods of data collection. Postpartum women who had brought their children for the second dose of measles vaccine between 18 and 24 months were sampled Participants were interviewed using structured questionnaires, data was collected about their socio-demographic characteristics, fertility, knowledge, use, and access to contraceptives. Chi square tests were used to determine the relationship between uptake of postpartum family planning and: socio demographic characteristics, contraceptive knowledge, use access and fertility. Qualitative data collection included focus group discussions (FDGs) with mothers and in-depth interviews with service providers Information was obtained from mothers’ regarding their perceptions on family planning methods, use, availability, access and barriers to uptake and key informants’ views on family planning counseling practices and barriers to uptake of family planning

**Results:**

More than three quarters (86.3%) of women used contraceptives within 1 year of delivery, with government facilities being the most common source. There was a significant association (*p* ≤ 0.05) between uptake of postpartum family planning and lower age, being married, higher education level, being employed and getting contraceptives at a health facility. One third of women expressing no intention of having additional children were not on contraceptives. In focus group discussions women perceived that the quality of services offered at the public facilities was relatively good because they felt that they were adequately counseled, as opposed to local chemist shops where they perceived the staff was not experienced.

**Conclusion:**

Contraceptive uptake was high among postpartum women, who desired to procure contraceptives at health facilities. However, there was unmet need for contraceptives among women who desired no more children. Government health facility stock outs represent a missed opportunity to get family planning methods, especially long acting reversible contraceptives, to postpartum women.

**Electronic supplementary material:**

The online version of this article (doi:10.1186/s12889-017-4510-6) contains supplementary material, which is available to authorized users.

## Background

Postpartum women are at a high risk of unplanned pregnancies, especially in the first year after delivery [1]. Adoption of postpartum contraceptives leads to not only a reduction in unplanned pregnancies, but also improves maternal and child well-being [2], since short birth intervals of less than 15 months are associated with adverse pregnancy outcomes: induced abortions, miscarriage, preterm births, neonatal and child mortalities, still births and maternal depletion syndrome [3–5].

During the postpartum period, there are multiple contacts between women and healthcare providers when women are seeking child immunization services, yet the unmet need for contraception is still high [6, 7]. A demographic health survey of four countries (2001) found that only 25% of women in Kenya had adopted postpartum family planning (PPFP) by six months, and 35% at one year [8]. Studies have shown that the need for contraceptives varies during a woman’s reproductive years, but demand is highest during the postpartum period [9, 10].

We do not know why there is a low uptake of family planning amongst postpartum women despite their multiple contacts with healthcare providers in health facilities.

Despite these interventions, unmet need for family planning among postpartum women remains high [11, 12]. Amixed methods study was designed to understand characteristics of postpartum women, correlates of family planning use, and the role of health providers in contributing to uptake of postpartum family planning. Our goals were to estimate the prevalence of postpartum family planning use in a rural hospital in Kenya (Kisii Level 5 Hospital) and to examine the factors that influence adoption of contraceptives among postpartum women.

## Methods

### Study design

This cross sectional study was conducted at maternal and child health clinics at Kisii level 5 hospital. This hospital is the largest government owned facility in the county of approximately 1.2 million population residing in the following 9 sub counties Kitutu Chache North, Kitutu Chache South, Nyaribari Masaba, Nyaribari Chache, Bomachoge Borabu, Bomachoge Chache, Bobasi, South Mugirango and Bonchari. The respondents were randomly selected from the clinic. Structured questionnaires were used to collect quantitative data and focus group discussions and in-depth interviews to gather qualitative data.

### Recruitment

Between April and May 2015, we recruited 365 mothers who had brought their children aged between 18 and 24 months for the second dose of measles vaccine at the maternal and child health clinics. Mothers whose children were aged below 12 months and more than 24 months were excluded from the study. Family planning clinic staff were also recruited for key informant interviews.

### Study procedures

At enrollment, we administered coded questionnaires to mothers, and collected information on maternal socio-demographic characteristics, knowledge (type of family planning known to the mothers), use (type of family planning used during postpartum period) and access (sources of contraceptives) to selected contraceptive methods (see Additional file 1). Mothers self-reported their contraceptive use. We defined postpartum family planning as being the uptake of modern contraceptives within a year of delivery. Research Assistants purposively recruited a subset of 20 participants who met the study criteria to participate in FGDs (see Additional file 2). We conducted two FGDs with each FGD having 10 mothers. Each FGD lasted between 45 and 60 min. Demographic characteristics of mothers who participated in the FGDs were captured. The FGD guide focused on mothers’ perceptions of FP methods, use, availability, access and barriers to uptake. Three KII were purposively recruited but one declined. The interviews were based on a discussion guide that covered availability of contraceptives, training and staffing of service providers, barrier and general uptake of family planning (see Additional file 3). Both key informant interviews (KII) and FGDs were moderated by the principal investigator, with the help of a research assistant who took notes and kept time. The KIIs were conducted in English while FDGs were conducted in both Swahili and English and the conversations were digitally recorded.

### Statistical analysis

Descriptive Statistics as percentages for the categorical variables are shown for selected predictors. (Table 1) Bivariate analysis were performed to examine the relationship between PPFP and the selected predictors. We used chi square tests to evaluate the relationship between the dependent variable (uptake of postpartum family planning) and the key independent variables (contraceptive knowledge, use, access and fertility). *P* value <0.05 was considered to be statistically significant. The tape recorded discussions and interviews were transcribed and translated into English. Manual content analysis was carried out and aligned to the main study themes. The relevant quotes have been highlighted in the text to illustrate the major findings.

**Table 1:** Socio-demographic characteristics of postpartum women

Factor	Classification	Frequency	Percent
Age	≤ 19	17	4.7
	20–24	165	45
	25–29	130	36
	30–34	25	6.8
	35–39	13	3.5
	40 and above	15	4.1
Marital Status	Single	47	12.8
	Married	287	78.4
	Separated/Divorced /Widowed	31	8.4
Education	No formal education	5	1.4
	Primary Level	29	7.9
	Secondary Level	185	50.5
	Tertiary Level	145	39.8
Employment Status	Not Employed	154	42.1
	Self Employed	128	35
	Salaried Employment	83	22.7
Religion	Catholic	100	27.4
	Protestant	107	29.2
	Muslim	5	1.4
	Seventh Day Adventist	153	41.9
Number of children	1–2 children	298	81.6
	3–4 children	49	13.4
	More than 5 children	18	5.0
Contraception Use (y/n)	Yes	317	86.8
	No	48	13.2
Method used	COCs	55	15.1
	DMPA injections	102	27.9
	Implanon	52	14.2
	IUCDs	88	24.1
	Male condoms	21	5.1

## Results

### Participant characteristics

We approached 1500 women at the maternal and child health clinics who had brought their children for immunization. 1085 were not eligible as they had children below 12 months of age or above 24 months. 50 mothers who met the study criteria declined to participate in the study, leaving 365 women who met the criteria and verbally consented to the study. The majority of the mothers (45%) were between 20 and 24 years of age; adolescents below 19 years were 4.7%; 78.4% of the women were married. Of women interviewed, 50.5% had completed secondary education while 39.8% had tertiary level education and above. On employment status, 42.1% of the women were not employed, whereas 22.7% were in salaried employment and 35% were self-employed. Respondents described their religion as 41.9% Seventh Day Adventists, 29.2% Protestants, 27.4% Catholics and 1.4% Muslims.

We found that 86.3% women used a family planning method within one year of delivery. The proportions of women using the various methods of contraceptives were: 27.9% used injectable contraceptives (depot medroxyprogesterone acetate, DMPA), 24.1% copper intrauterine contraceptive device (IUCD), 15.1% combined oral contraceptive pills (COCs) and 14.2% levonorgesterol containing implants (Implanon). Nearly all of the respondents (98%) reported obtaining information on contraceptives from health care workers with COCs being the most known (96.7%) type of contraceptive method. Government facilities were the most popular providers of IUCDs (99%), implanon (74%) and COCs (67%) see Table 1.

### Socio-demographic factors and postpartum family planning adoption

Compared to women who were not using contraception, women using PPFP were younger and more likely to be married (*p* < 0.01). These findings are consistent with those of the key informant interviews and FGDs, where it was confirmed that most of the women attending maternal and child health clinics were young and their preferred method of contraception was DMPA injection as stated by one of the key informants.

*“Majority of our clients are young women less than 30 years of age. Many of the mothers come to the clinic with a fixed mind of getting injections”.*

The most apprehended side effects were bleeding, high blood pressure, palpitations and weight gain. One of the women commented:

*“I started using injections when my baby was 6 months old, but because of heavy bleeding I had to stop using”.*

One of the women in the FGDs commented:

*“When you want something you already know about it. Three months after my child’s delivery I came to the clinic knowing that I wanted to get an injection. I was taught about the other methods of family planning. It was like they were telling me to switch to another method indirectly since they had run out of injectable contraceptive. I told them that I will go home and make up my mind but I never returned to the facility”.*

Choice of contraceptive methods varied with level of education. Women with secondary education and above, were more likely to choose long term methods as opposed to those with primary education and below (*p* = 0.02). All the respondents in the FGDs were familiar with modern methods of contraception. They cited some of the barriers to why women do not adopt family planning as side effects, myth and misconceptions, spousal refusal, religion and lack of education. One of the women said:

*“my neighbor has many children and she is still pregnant. She has not gone to school and has no job while the husband drinks daily. I try to talk to her about family planning but she said that the bible has said we fill the earth with children”.*

In the KII stated that:

*“women are ready to take up family planning although myths like the belief that IUCD travels to the heart hinder uptake of these methods”*

Women’s employment status significantly predicted PPFP uptake. Women who were in employment were more likely to use contraceptives. Of those who indicated that they had not adopted PPFP, 7.7% were unemployed (*p* < 0.01) (see Table 2). Religious affiliation was associated with adoption of PPFP. Seventh Day Adventists were more likely to adopt family planning method. (*p* = 0.01). This finding is consistent with the FGDs where the women who prescribe to Seventh Day Adventist confirmed that they were taught family planning in church to enable them to space their children.

**Table 2:** Correlates of postpartum family planning

Factor		Used PPFP within a year % (*n*)		*P* value
		Yes	No	
Age	≤ 25	40.9 (149)	8.8 (32)	0.02
	26–30	34.8 (127)	2.7 (10)	
	31–35	6.8 (25)	1.1 (4)	
	≥36	4.4 (16)	0.5 (2)	
Marital status	Not in union	15 (55)	5.5 (20)	˂0.01
	In union	71.8 (262)	7.7 (28)	
Education	No education	1.1 (4)	0.3 (1)	0.02
	Primary school	6.6 (24)	1.7 (6)	
	Secondary education	41.6 (152)	7.4 (27)	
	Tertiary education	37.7 (138)	3.6 (13)	
Employment status	Not employed	33.4 (122)	7.7 (28)	˂0.01
	Self employed	31.8 (116)	3.6 (13)	
	Salaried employment	21.6 (79)	1.9 (7)	
Number of children	1–2	71.2 (260)	10.4 (38)	0.120
	3–4	12 (44)	1.4 (5)	
	5 and above	3.6 (13)	1.4 (5)	
Planning to have more children in future	Yes	50.1 (183)	4.4 (16)	˂0.01
	No	21.6 (79)	4.1 (15)	
	Depends on my husband	7.9 (29)	1.9 (7)	
	I don’t Know	7.1 (26)	2.7 (10)	
Waiting duration before the conception of another child	< 2 years	12 (36)	3 (9)	˂0.01
	2 to 4 years	50 (142)	2 (6)	
	I don’t know	23 (67)	7 (21)	

One woman commented:

*“In our parish we are encouraged to use contraceptives to plan our families”.*

### The effect of health system factors on contraceptive use among postpartum women

Uptake of PPFP was significantly associated with counseling of women on various types of contraceptives (*p* < 0.01). These findings are in agreement with the FGDs, where a majority of the women endorsed that the quality of services offered was good, but acknowledged a major challenge: frequent stock outs at the facility.

*“I had stopped using injection and I wanted to change to coil (IUCD), I came to the hospital and I was told that they had run out of coil. I was told to come back after a month. Unfortunately, I became pregnant when I was waiting to go back to the hospital*”.

Frequent stock outs made women go to the chemists for supplies, even though they felt the counseling services at the chemists to be inadequate. This quote illustrates women’s perceptions of services offered by various facilities.

“*I was told by my friends that getting injections from chemists is bad. It’s not everyone who owns a chemist that is qualified. You may find that some of them lack experience. It’s better to go to a public facility which is safe”.*

Key informant interviews confirmed frequent stock outs in the facility especially for DMPA injection and contraceptive implant.

*“We run out of stock. Currently we don’t have Depo provera and implanon, but counseling on various family planning methods still continues”.*

### The effect of fertility on contraceptive use among postpartum women

Parity was not a predictor of PPFP utilization (*p* = 0.12). Women who had adopted PPFP were more likely to report planning to have more children in the future. A significant difference was observed between the intention to have more children in future and the type of contraceptive used (*p* < 0.01). DMPA injections, contraceptive implants and IUCDs were more likely to be used by women planning to have children in the near future. Women who did not report wanting additional children reported the following contraceptive methods: male condoms (9.5%), DMPA injection (19.6%), COCs (30.9%).

Women in the FGDs shared concerns raised by their spouses on the use of contraceptives.

*“I started using injection for family planning without consulting my husband. When he later found out he was happy because he was not consulted”.*

*“I have 3 children, I think they are enough but my husband says I can only use family planning when he thinks the children are enough”.*

The KII commented:

*“we are trying to encourage men to bring their women to the clinic for family planning by ensuring that women who come with their spouses are not made to queue like the rest and are served promptly”.*

However 34.1% of the women with no intent to have more children were not using any contraceptive methods. In this study, 57% of the women using condoms did not know whether they wanted to space and others deferred the decision on fertility intention to their husbands. Similarly, 27% of those using DMPA injections and 16% of those using COCs had not decided whether they wanted future children see Table 2.

## Discussion

In this study, more than three quarter (86.3%) of the respondents were found to have adopted postpartum contraception. This finding contrasts with some recent studies in Kenya. For instance, the Nairobi Urban Health and Demographic Surveillance System (NUHDSS, 2011) indicated that while resumption of sex occurs quite early (50%) by the third month, relatively few women initiate contraceptive use during the first six postpartum months [13]. This variation between the present study and other studies in sub Saharan Africa [14], can be explained by the difference in methodologies adopted in the highlighted studies. Whereas, this was a cross sectional study of postpartum women attending a health facility in a quasi-rural setting the NUHDSS was a longitudinal study which recruited participants at the household level in a primarily urban setting. As such, women who are not regular attendees of postpartum clinics participated.

In this study, use of COCs, DMPA injections and condoms was relatively high. This finding reflects national trends that show that women prefer modern contraceptives (53%) to the traditional methods (5%) [15]. This finding is also consistent with a recent Demographic Health Survey (DHS) analysis of 21 low and middle income countries, which showed that women who adopt modern contraceptive methods postpartum are likely to opt for short term hormonal methods [1]. In our study, as in prior studies, married women were more likely to adopt family planning compared to their unmarried counterparts [16, 17]. This could be because married women are exposed to frequent sexual activities [18].

Adoption of PPFP was high among women with high post primary education, this is in keeping with other studies [16, 17, 19], and there was a significant association between higher education level and contraceptive awareness. This finding is consistent with a study done in Ethiopia, where it was observed that women with higher education level were more likely to adopt a family planning method [20].

Our findings showed that condoms were most popular among women with primary level of education and below, whereas highly effective long-acting methods such as contraceptive implants and IUCDs were more common among women with secondary education and above. See Table 3. Women’s employment status is positively associated with PPFP in our study. Studies done elsewhere have revealed a similar relationship between employment and contraceptive use [18, 21, 22]. This may be explained by the fact that economically empowered women can access facilities and can afford the cost of contraceptives more easily than women who do not have a source of income.

**Table 3:** Relationship between level of education and family planning methods

		Primary Education and below	Secondary School	Tertiary education
Male condoms	Count	6	11	4
	% FP Method	28.6%	52.4%	19.1%
IUCD	Count	9	34	45
	% FP Method	10.2%	38.6%	41.2%
Implant	Count	4	23	24
	% FP Method	7.9%	45.1%	47%
Injection	Count	6	57	39
	% FP Method	5.9%	55.9%	38.2%
Female Sterilization	Count	0	4	1
	% FP Method	0.0%	80%	20%
Oral pills	Count	7	27	21
	% FP Method	12.7%	49.1%	38.2%
None	Count	3	22	16
	% FP Method	7.5%	52.50%	40%

This study established that younger women were the most avid users of postpartum contraception especially between age 19–24 years. Previous studies have demonstrated a significant relationship between age and contraceptive use [17, 23]. A DHS analysis of 21 countries showed that a majority of women who use contraceptives are aged between 20 and 34 years [21]. This may be because women in the higher age groups may assume that they are not fecund and therefore, have no need for contraception, or they are not accessing the facility so we did not capture them in this study. It may also be because the women in higher age groups are more socially conservative and declined to participate in the interviews.

A majority of women at the clinic obtained contraceptives from government hospitals. In this facility, we established that the quality of care is relatively good because the participants felt they got adequate counseling on family planning. However, the impact of these excellent services was diminished due to frequent stock outs at the public facilities, long waiting queues and staff shortage appeared to be pushing women to use private pharmacy services, where women felt that they were not adequately counseled. The demand for contraception among the respondents driven by information disseminated by healthcare workers was not matched by efficient supply which resulted in frequent stock outs.

In addition, some of the respondents had their contraceptive implants and DMPA injections administered by chemists contrary to the National Family Planning Guidelines for Service Providers, 2010 [24]. Previous studies have demonstrated that a woman’s decision to adopt a family planning method is strongly influenced by how she perceives the quality of health care service provided [17, 25]. This conclusion has been corroborated by a qualitative study done in the informal settlements of Mathare, Kenya. The women were more confident of the family planning services offered at public facilities than in private institutions, perceiving the latter to have prioritized profits over safe medical practice [12].

Unlike a population based study in Uganda which demonstrated a positive association between a woman’s number of living children and her likelihood of using PPFP, parity was not a predictor of PPFP uptake in this study [19]. Some women expressing no intention to have more children or those with spacing plans of more than two years were either not on any contraceptives, or opted for short term hormonal methods. This finding is consistent with a study conducted in five low income countries by Pasha et al., which showed that the uptake of long acting reversible contraceptives among postpartum women is low [14]. Some women expressing no desire to have more children in the future were on short term methods of contraceptives. This suggests that there is room for improvement for counseling services even in government facilities, to promote long acting methods. Further research should investigate reasons why uptake of long term contraceptive methods is low among postpartum women.

Our study had some limitations. Our results may not be generalisable as we conducted the study among women attending maternal and child health clinics in a rural area. Our study also used a non random selection of participants. We may have missed some women who did not come to the clinic for immunizations, or who received their health care in other settings. Our FDGs took place in the hospital and as such the women may have viewed the principal investigator as part of the healthcare providers, which may have prevented them from answering questions as freely as if they were offsite.

## **Conclusion**s

Among postpartum women attending a government health clinic, we found that uptake of FP at one year postpartum was high and was strongly associated with marital status, higher education level, younger age, being employed and getting contraceptives at the clinic. Public health campaigns targeting women with low levels of education should be the focus of future efforts to improve uptake of PPFP. Policy makers should work with various family planning stakeholders to make sure that postpartum women who do not want additional children are counseled to use long acting contraceptives. The study also reveals a gap in the skills and knowledge of family health care workers especially those in private facilities and presents an opportunity for training them in family planning methods. Finally, we identified that stock outs at the facility were consistently hindering motivated women from getting the method that they wanted, and were increasing risk of unplanned pregnancies in the postpartum period. In our sample, government family planning clinics are trusted and attended by women in the community; therefore the simplest way to increase uptake of PPFP is to ensure that they are always stocked with the desired contraceptives.

## Additional files

Additional file 1: Respondents’ Questionnaire. (DOCX 16 kb)

Additional file 2: Focus Group Discussion Guide. (DOCX 12 kb)

Additional file 3: Key Informant Interview Discussion Guide. (DOCX 13 kb)

## Abbreviations

COCs: Combined oral contraceptive pills
DHS: Demographic health survey
DMPA: Depot medroxyprogesterone acetate injection
ERC: Ethics and research committee
FDG: Focus group discussion
FP: Family planning
IUCD: Intra uterine contraceptive device
KNH/UoN: Kenyatta National Hospital/University of Nairobi
NUHDSS: Nairobi Urban Health and Demographic Surveillance System
PPFP: Postpartum family planning
SPSS: Statistical package for the social science
