# Supplementary material for: Determinants of contraceptive use among postpartum women in a county hospital in rural KENYA
Source: BMC Public Health. 2017 Jun 29;17:604. doi: 10.1186/s12889-017-4510-6 (PMC5492366; doi:10.1186/s12889-017-4510-6)
Supplement: Supplementary file 1 — Respondents’ Questionnaire. (DOCX 16 kb) [file 12889_2017_4510_MOESM1_ESM.docx]

**Respondents’ Questionnaire**

Interviewer:

Participant’s Identification number:

Date: Time:

Thank you very much for agreeing to participate. Am going to start asking the questions:-

**Socio-demographic Information**

1. How old are you?............. years
2. How old is your child?.......(in months)
3. What is your current marital status? (Circle one answer only)
4. Married
5. Living with partner
6. Separated
7. Divorced
8. Widowed
9. Single
10. If yes, how long?.......(in years)
11. What is the highest level of your education? (Circle one answer only)

1. No education

2. Primary school dropout.

3 Primary school completed

4 Secondary school drop out

5 Secondary school completed

6 College/University dropout

7 College/University completed

8 Post graduate degree

9 Refused

1. Which of the following best describes your employment status over the past 12 months? (Circle one response)
   1. Government employee
   2. Non-government employee
   3. Self-employed
   4. Non-paid
   5. Student
   6. Housewife
   7. Casual worker

**Social cultural information**

1. What is your religion? (Circle one answer only)
2. Catholic
3. Protestant
4. Muslim
5. Seventh Day Adventist
6. Others…………………..(Specify)
7. Which family planning methods do you know? (Circle all those mentioned)
8. Oral pills
9. Female condom
10. Male condom
11. IUCD
12. Implant
13. Injection
14. Female Sterilization
15. Male sterilization
16. Natural family planning
17. Lactational Amenorrhoea (LAM)
18. Where did you get information on family planning? (Circle all those mentioned)
19. Television
20. Radio
21. Newspaper/magazines
22. Health care workers
23. Friends
24. Spouse
25. Internet
26. Others…………………….…(please specify)
27. Did you use any form of family planning within one year of delivery?
    1. Yes
    2. No
28. If yes, at how many months did you start using?
29. Record months………
30. I don’t know
31. Not applicable
32. Which method of family planning did you use? (Circle all those mentioned)
33. Oral pills
34. Female condom
35. Male condom
36. IUCD
37. Implant
38. Injection
39. Female Sterilization
40. Male sterilization
41. Natural family planning
42. Lactational Amenorrhoea (LAM)
43. None
44. Was your spouse involved in the selection of family planning method?
45. Yes.
46. No.
47. Not applicable
48. Where did you get the family planning method you were using? (Circle one answer)
49. Private facility
50. Government health facility
51. Faith based organization
52. Chemists / Pharmacy
53. Others………………….(please specify)
54. None

**Health system factors**

During your visits to the hospital within one year of delivery, did the health care worker

1. Provide information on different methods of family planning?
2. Yes
3. No
4. I don’t know
5. Ask about your family planning preference?
6. Yes
7. No
8. I don’t know
9. Help you select a family planning method?
10. Yes
11. No
12. I don’t know
13. Talk about possible side effects?
14. Yes
15. No
16. I don’t know.
17. What are some of the side effects discussed with you? (Circle all those mentioned)
18. Vision loss or blurring
19. Severe abdominal pain
20. Severe leg pain
21. Late period
22. Heavy periods
23. Jaundice
24. Neurological signs
25. None
26. Others …………………………
27. Tell you what to do if you had side effects with family planning
28. Yes
29. No
30. I don’t know
31. Did they give you a return date?
32. Yes
33. No
34. I don’t know

**Behavioral Factors**

1. How many living children of your own do you have?
2. Record number…….
3. Don’t know
4. Would you like to have more children in the future? (Circle one answer)
5. Yes
6. No
7. Depends on husband
8. I don't know
9. How long would you like to wait from now before the conception of another child? (Circle one response)
10. Less than one year.
11. One to two years
12. More than two years
13. I don’t know

THANK YOU FOR YOUR TIME
