# Supplementary material for: Determinants of contraceptive use among postpartum women in a county hospital in rural KENYA
Source: BMC Public Health. 2017 Jun 29;17:604. doi: 10.1186/s12889-017-4510-6 (PMC5492366; doi:10.1186/s12889-017-4510-6)
Supplement: Supplementary file 2 — Focus Group Discussion Guide. (DOCX 12 kb) [file 12889_2017_4510_MOESM2_ESM.docx]

## Focused Group Discussion Guide

1. What consideration do you make when choosing family planning?
2. What are your opinions on family planning postpartum?
3. What are the barriers that hinder you from taking family planning?
4. What do you think can be done to remove these barriers?
5. What is the availability of family planning methods?
