# Supplementary material for: Determinants of contraceptive use among postpartum women in a county hospital in rural KENYA
Source: BMC Public Health. 2017 Jun 29;17:604. doi: 10.1186/s12889-017-4510-6 (PMC5492366; doi:10.1186/s12889-017-4510-6)
Supplement: Supplementary file 3 — Key Informant Interview Discussion Guide. (DOCX 13 kb) [file 12889_2017_4510_MOESM3_ESM.docx]

# APPENDIX VI

## Key Informant Interview Discussion Guide

1. How many ……………. (Name of cadre which the interviewee is in-charge of) are deployed in this clinic?(MCH)

| **Qualification** | **Number** |
| --- | --- |
|  |  |

1. How many ………… have been trained on family planning guidelines as per the National Family Planning Guidelines for Service Providers?

Number:

1. How many ………… are on duty at any given clinic day?

Number:

1. What are your experiences in family planning in postpartum women?
2. What challenges do you face when it comes to uptake of postpartum family planning?
3. What are your experiences with family planning counseling practices?
